# Analysis of 3D Channel Current Noise in Small Nanoscale MOSFETs Using Monte Carlo Simulation

**DOI:** 10.3390/nano14161359

**Published:** 2024-08-18

**Authors:** Wenpeng Zhang, Qun Wei, Xiaofei Jia, Liang He

**Affiliations:** 1 School of Physics, Xidian University, Xi’an 710071, China; 2 School of Advanced Materials and Nanotechnology, Xidian University, Xi’an 710071, China

**Keywords:** 3D Monte Carlo simulation, channel shot noise, thermal noise, gate-tunneling shot noise

## Abstract

As field effect transistors are reduced to nanometer dimensions, experimental and theoretical research has shown a gradual change in noise generation mechanisms. There are few studies on noise theory for small nanoscale transistors, and Monte Carlo (MC) simulations mainly focus on 2D devices with larger nanoscale dimensions. In this study, we employed MC simulation techniques to establish a 3D device simulation process. By setting device parameters and writing simulation programs, we simulated the raw data of channel current noise for a silicon-based metal–oxide–semiconductor field-effect transistor (MOSFET) with a 10 nm channel length and calculated the drain output current based on these data, thereby achieving static testing of the simulated device. Additionally, this study obtained a 3D potential distribution map of the device channel surface area. Based on the original data from the simulation analysis, this study further calculated the power spectral density of the channel current noise and analyzed how the channel current noise varies with gate voltage, source–drain voltage, temperature, and substrate doping density. The results indicate that under low-temperature conditions, the channel current noise of the 10 nm MOSFET is primarily composed of suppressed shot noise, with the proportion of thermal noise in the total noise slightly increasing as temperature rises. Under normal operating conditions, the channel current noise characteristics of the 10 nm MOSFET device are jointly characterized by suppressed shot noise, thermal noise, and cross-correlated noise. Among these noise components, shot noise is the main source of noise, and its suppression degree decreases as the bias voltage is reduced. These findings are consistent with experimental observations and theoretical analyses found in the existing literature.

## 1. Introduction

Transistors are indispensable to chip technology, with smaller gate sizes enhancing integration and performance. As the dimensions of transistors continue to shrink, devices become faster and circuits achieve higher densities, which has driven the rapid development of the semiconductor industry. Moore’s Law is a testament to this trend. With the progression of technology nodes, a variety of emerging materials and innovative design structures have been proposed [1,2,3,4], and transistors with features of 10 nm and below have been designed and implemented [5,6,7]. However, the miniaturization of device sizes also introduces new challenges, among which the issue of device noise has become one of the key factors limiting device performance. The metal–oxide–semiconductor field-effect transistor (MOSFET), as a pivotal component of modern integrated circuits, directly impacts the efficiency and reliability of the entire system. As device sizes decrease, the problem of channel current noise becomes increasingly severe. This type of noise not only compromises the integrity of signals but may also restrict the operating frequency and power efficiency of the device [8,9,10]. Therefore, a profound investigation into the characteristics of channel current noise in small-sized MOSFET devices is essential.

In small nanoscale MOSFETs, noise arises from irregular carrier motion owing to current spreading and drift. Specifically, shot noise results from the randomness of carriers crossing potential barriers [11]. In small nanoscale MOSFETs, a potential barrier exists in the channel, and carriers randomly cross this barrier at the source to enter the channel, generating channel shot noise. On the other hand, thermal noise arises from the random thermal motion of carriers in resistive elements. Field effect transistors function by modulating the resistance of the conductive channel. As carriers enter the channel, they undergo multiple scattering events under the influence of the applied electric field before being collected by the drain, resulting in thermal noise [12]. With ongoing miniaturization, the oxide layer thickness is reduced to a few atomic layers, facilitating gate-induced carrier tunneling. Some carriers may randomly tunnel through the oxide layer potential barrier into the channel, creating gate-tunneling shot noise. Concurrently, other carriers may interact with channel charges, resulting in cross-correlation noise [13,14,15]. Previous studies on channel current noise in nanoscale devices have focused mainly on thermal noise and suppressed channel shot noise [9,16,17], often neglecting gate-tunneling shot noise and cross-correlation noise. Therefore, it is crucial to identify and analyze all noise components in small-scale nano-MOSFET devices.

Traditional semiconductor device parameter simulation tools have typically used the drift–diffusion (DD) and hydrodynamic (HD) models. However, these tools have limitations when simulating short-channel nanoscale devices, particularly in evaluating noise performance. In comparison, the Monte Carlo (MC) [18,19,20,21] simulation method distinguishes the noise characteristics of short-channel devices from those of long-channel devices. It effectively captures the dynamics of carrier motion in semiconductor devices and provides a relatively straightforward simulation process [22,23]. Therefore, the MC method is better suited for the noise simulation analysis of small-size nanoscale devices, and it has already yielded reliable research results in simulating noise mechanisms [24,25]. Previous studies employing the MC simulation method have often focused on the 2D noise analysis of large-sized devices. Therefore, this study uses the MC method to simulate channel current noise in small-size MOSFETs with a 10 nm channel length. By developing a 3D channel current noise simulation framework and establishing relevant device simulation parameters, this study obtains the 3D potential distribution along the device channel length and the MOSFET current–voltage characteristic curve. Furthermore, by extracting the channel current noise power spectral density (*S*_id_) from current fluctuations over a unit time step, the relationship between channel current noise and full shot noise is determined. Finally, in this study, we investigate the influence of various parameters on channel current noise, including gate–source bias voltage (*V*_GS_), drain–source voltage (*V*_DS_), temperature (*T*), and channel doping density (*N*_CH_).

## 2. Monte Carlo Simulation

The MC simulation method is based on principles of stochastic probability and mathematical statistics. It entails constructing random models and using computational tools to generate random numbers, which are then used to establish statistical distributions and sample these models. This process enables the numerical solution of the required problem [26]. Because the MC method involves simulating objects with inherent randomness, and because noise results from the irregular random motion of carriers in the device, it is highly effective for studying the noise characteristics of the device.

### 2.1. Scattering Mechanisms

**Figure 1:**
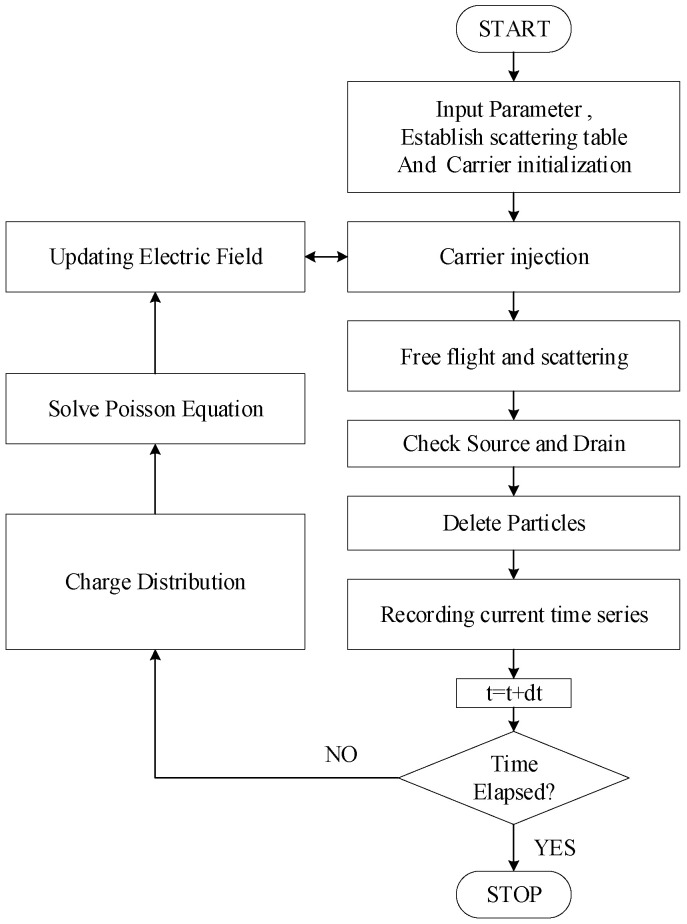
Flowchart of particle−based device simulator.

In MOSFET devices, carriers in the channel region undergo free flight and collisions influenced by an applied electric field. The basic flowchart of the MC simulation is depicted in Figure 1. During simulation, the scattering table encompasses three types of scattering mechanisms: ionized impurity scattering, acoustic phonon scattering, and optical phonon scattering [22,27]. The scattering probability of ionized impurity scattering is expressed as follows:

(1) $$p\left(\varepsilon \right)=\frac{2^{5/2}\pi ne^{4}Z^{2}}{\kappa^{2}\varepsilon_{\beta }^{2}m_{d}^{1/2}}\cdot \frac{\varepsilon^{1/2}}{\left(1+4\varepsilon /\varepsilon_{\beta })\right.}$$

(2) $$\varepsilon_{\beta }=\frac{\hbar^{2}\beta^{2}}{2m_{d}}$$

(3) $$m_{d}={\left(m_{l}m_{t}^{2}\right)}^{1/3}$$

where *Z* is the concentration of ionized impurity doping, κ is the dielectric constant of the material, n is the electron density, and $m_{d}$ is the effective mass of the density of states.

(4) $$p(\varepsilon )=\frac{\sqrt{2}\cdot m^{\frac{3}{2}}k_{B}T\sum^{2}}{\pi \hbar^{4}c_{l}}\cdot \varepsilon^{\frac{1}{2}}(1+\alpha \varepsilon {)}^{\frac{1}{2}}$$

The scattering probability of acoustic phonons is expressed by Equation (4), where Σ is the material defect potential (the potential energy arising from the interaction between phonons and lattice defects), $c_{l}$ is the elastic constant, α is the non-parabolic factor, $k_{B}$ is the Boltzmann constant, and T is the lattice temperature.

(5) $$p\left(\varepsilon \right)=\frac{m^{3/2}D_{0}}{\sqrt{2}\pi \rho \hbar^{2}\left(\hbar \omega_{0}\right)}\left(N_{q}+\frac{1}{2}\pm \frac{1}{2}\right)\cdot \sqrt{\varepsilon \pm \hbar \omega_{q}}\cdot \sqrt{1+\alpha \left(\varepsilon \pm \hbar \omega_{q}\right)}\cdot \left(1+2\alpha \left(\varepsilon \pm \hbar \omega_{q}\right)\right)$$

(6) $$N_{q}=\frac{1}{exp\left(\frac{\hbar \omega_{q}}{k_{B}T}\right)-1}$$

The optical phonon scattering probability is expressed by Equation (5), where $D_{0}$ is the optical deformation potential, $N_{q}$ is the Bose–Einstein distribution function, describing the distribution of bosons in thermal equilibrium, which can be used to calculate the phonon occupation number. $\hbar \omega_{q}$ is the phonon energy. The terms +/− indicate the absorption and emission parts, respectively.

### 2.2. Device Simulator Detail

Most of the computational time in the MC simulation is dedicated to managing random flight and scattering of charge carriers, specifically handling their transport. As depicted in Figure 1, first, injected carriers undergo free flight and scattering in the applied electric field, resulting in a new electron distribution. Subsequently, using the carrier information matrix, the real-space and K-space coordinates of the carriers are determined, and carriers exiting the channel region of the device are removed. Next, based on the positions of carriers, the dynamic Poisson equation is resolved to update the electric field, preparing for the next iteration of carrier injection and scattering. This cycle repeats until the predetermined total simulation time is reached. During this process, a three-dimensional electron injection model is used for the source and drain regions [28]. This process can effectively take into account the Fermi and Coulomb correlations between charge carriers. 

After completing the simulation, the channel current sequence of the device is obtained based on the simulation time interval, and the power spectral density of the channel current noise is calculated. Equation (7) is used to calculate the drain output current [19].

(7) $$i_{d}\left(t\right)=\frac{\sigma }{L}\sum_{i=1}^{M}v_{i}\left(t\right)$$

Here, *L* is the length of the computational region, *M* is the number of charge carriers in the region, *σ* is the charge carried by each charge carrier, and $v_{i}\left(t\right)$ is the instantaneous velocity of the charge carrier obtained from the simulation. After calculating the instantaneous drain output current $i_{d}\left(t\right)$ of the device, the steady-state current $I_{d}$ can be obtained by averaging the instantaneous current. Because noise is a random signal, its magnitude is characterized by calculating its power spectral density. The power spectral density is derived from the autocorrelation function, and the noise power spectral density of the current fluctuations at the transistor port is extracted through MC simulation. The instantaneous current fluctuations induced by the noise of the device are represented as follows [29]:

(8) $$i_{nd}\left(t\right)=i_{d}\left(t\right)-I_{d}$$

The formula for calculating the channel current noise power spectral density of the device is as follows:

(9) $$S_{id}=\frac{2}{\pi }\int_{0}^{\infty }C_{d}\left(\tau \right)cos\left(\omega \tau \right)d\tau$$

In the expression, $C_{d}\left(\tau \right)$ is the autocorrelation function of the instantaneous current fluctuations of device, $i_{nd}\left(t\right)$, and its formula is given by the following:

(10) $$C_{d}\left(\tau \right)\leq i_{nd}\left(t\right)i_{nd}\left(t+\tau \right)\geq \frac{1}{T}\int_{0}^{T}i_{nd}\left(t\right)i_{nd}\left(t+\tau \right)dt$$

The typical expression for shot noise is given by the following:

(11) $$S_{id}=\gamma 2q\left(I_{d}\right)$$

where q is the elementary charge of an electron, $I_{d}$ is the drain–source current, and γ is the suppression factor for shot noise. When γ = 1, Equation (11) is referred to as full shot noise; when γ < 1, Equation (11) is referred to as suppressed shot noise.

The energy band structure of silicon is modeled using a non-parabolic model with the model expression:

(12) $$\varepsilon \left(k\right)\left(1+\alpha \varepsilon \left(k\right)\right)=\frac{\hbar k^{2}}{2m^{*}}$$

The nature of noise in the channel region of the device can be identified by comparing the noise power spectral density of the device channel current with the full shot noise. Subsequently, the relationship between noise power spectral density (*S*_id_) and gate–source bias voltage (*V*_GS_), source–drain bias voltage (*V*_DS_), temperature (*T*), and channel doping density (*N*_CH_) is analyzed.

## 3. Results and Discussion

### 3.1. Device Structure

**Figure 2:**
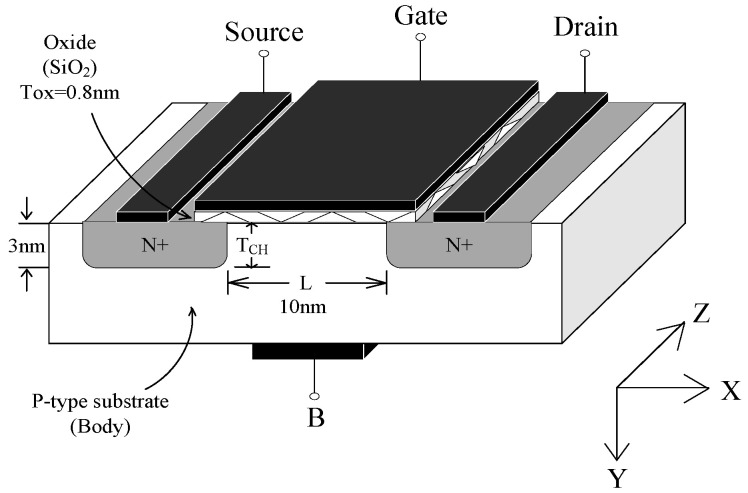
The schematic diagram of the simulation structure for a MOSFET.

Based on the MC simulation process described in the previous section, the relevant simulation parameters are configured to simulate carrier transport in MOSFET devices with a channel length *L* = 10 nm. Figure 2 shows the structure of the 10 nm MOSFET device, with the oxide layer thickness (*T*_OX_) set to 0.8 nm and the channel thickness (*T*_CH_) set to 3 nm. The source and drain regions consist of ideally isotropic n-type silicon with a doping density *N*_D_ of 2 × 10^21^ cm^−3^, and the channel region is composed of low-doped *p*-type silicon with a doping density *N*_A_ of 2 × 10^17^ cm^−3^. Given the simulation focuses on the channel inversion layer, this study uses a densely meshed channel area with a 120 × 50 × 1 3D grid structure. Furthermore, the simulation time step Δt is set to 1 fs to accurately capture the rapid dynamic changes in the carriers at the nanoscale.

### 3.2. Physical Parameter

The physical parameters selected for the simulation procedure are shown in Table 1. These physical parameters include the scattering model parameters and energy band model parameters described in the previous section.

### 3.3. Simulation Program

**Figure 3:**
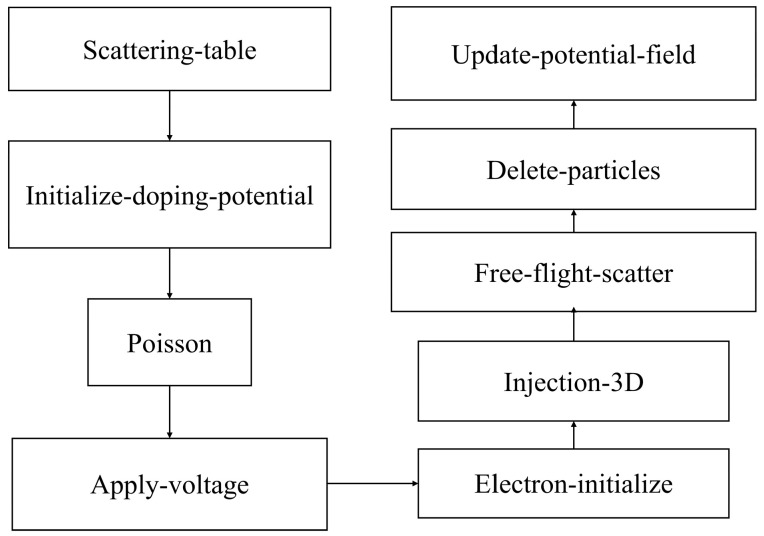
Schematic diagram of program structure.

With the established carrier transport flow, the algorithm is programmed to simulate, as shown in Figure 3, which is a schematic diagram of the program structure, and the sub-module functions are called through MATLAB to achieve the simulation of the 10 nm MOSFET. 

The functions of each module function are as follows:

Scattering-table: responsible for storing scattering types and calculating scattering rates.
Initialize-doping-potential: responsible for reading the input parameters and initializing the doping potential of the device.
Poisson: solving for the potential distribution in the channel area of a device.
Apply-voltage: applying external voltage conditions to the device.
Electron-initialize: initializes the carrier distribution and randomly generates carrier real space and K-space coordinates in the carrier information matrix.
Injection-3D: realization of source region carrier injection.
Free-flight-scatter: acts as the main program and handles carrier transfer.
Delete-particles: removes carriers from outgoing devices.
Update-potential-field: calculations update dynamic electric field and potential.

### 3.4. Static Tests

**Figure 4:**
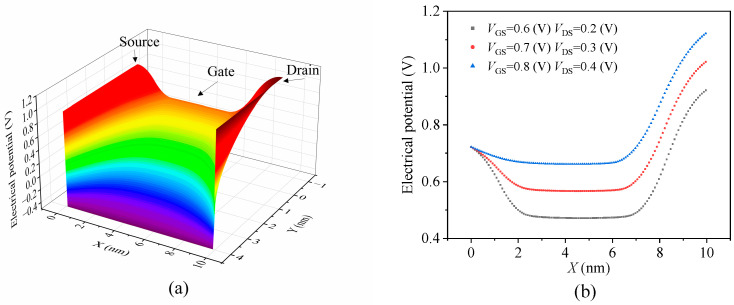
(**a**) The potential distribution map of the device along the channel is presented at a gate–source voltage of 0.7 V and a drain–source voltage of 0.3 V; (**b**) the two-dimensional potential distribution map of the device’s channel region surface direction under different bias voltages.

**Figure 5:**
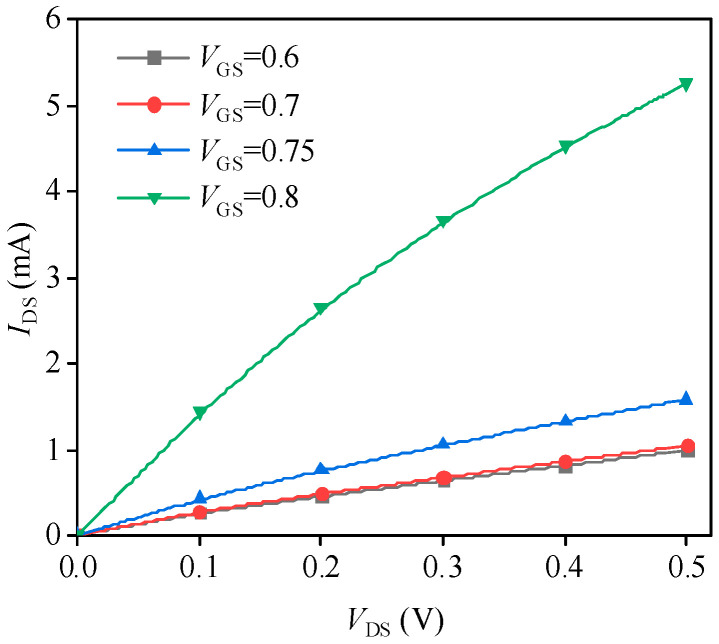
Simulation results of output I-V characteristics of 10 nm MOSFET.

Figure 4a illustrates the potential distribution along the 10 nm channel length direction when the device operates at a gate–source voltage of 0.7 V and a drain–source voltage of 0.3 V. The high doping of n-type silicon in the source and drain regions results in elevated potentials in these areas compared to the substrate. Simultaneously, applying the drain–source voltage further increases the potential at the drain, resulting in a strong electric field effect near the drain in the nanoscale MOSFET. The application of the gate voltage also induces an increase in potential at the device surface, resulting in a weak inversion state. Figure 4b represents a two-dimensional projection of the three-dimensional potential distribution on the device surface as shown in Figure 4a, illustrating the variation in surface potential under different bias voltages. Figure 5 presents the I–V curves of the transistor for different biases. As the gate–source voltage increases from 0.75 V to 0.8 V, there is a significant rise in the channel current. This increase occurs either because the device transitions from a weak inversion region to a strong inversion region, or because the higher gate–source voltage induces gate carriers to tunnel through the oxide layer and reach the channel, thereby forming a tunneling current. The simulated output characteristics of the device correspond with the inherent properties of MOSFETs, confirming the efficacy of the MC simulation process employed in this study.

**Figure 6:**
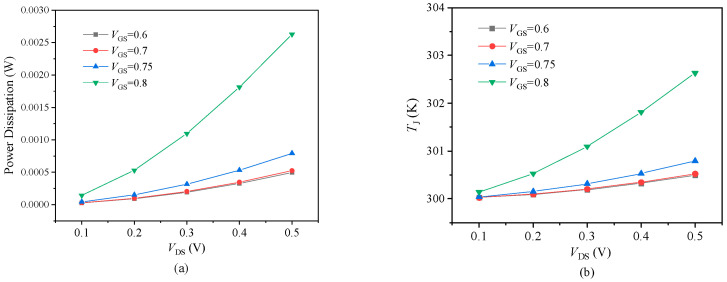
(**a**) Power dissipation at different gate−source biases; (**b**) variation in junction temperature with bias voltages.

Based on the outcomes of the static simulation tests, we conducted a further computational analysis of the power dissipation and junction temperature variation in the 10 nm MOSFET device with respect to the source–drain bias voltage, as depicted in Figure 6. As the voltage increases gradually, both power consumption and junction temperature demonstrate analogous variational trends. The power dissipation and junction temperature are maintained within a comparatively low range, aligning with the design principles of miniature devices.

### 3.5. The Impact of Bias Voltage on Channel Noise

**Figure 7:**
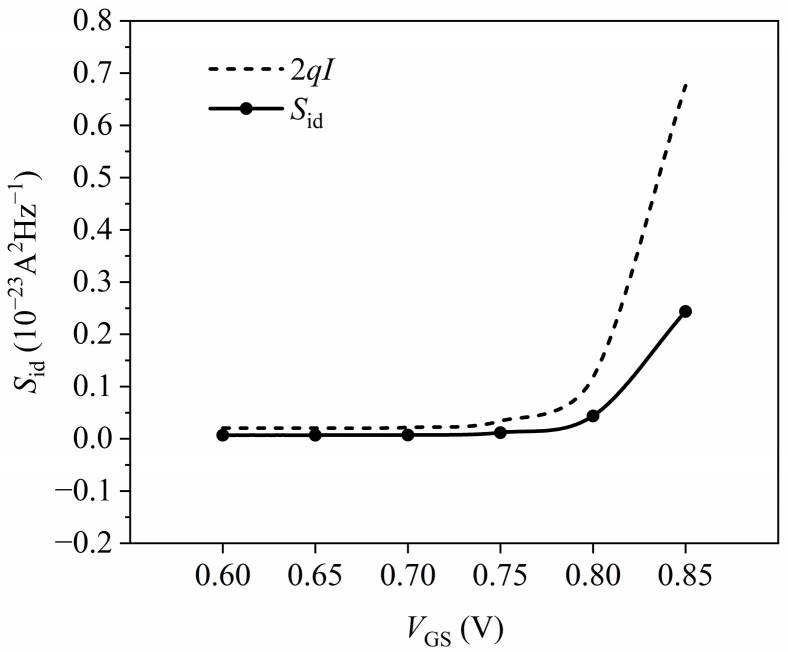
Relationship between simulation power spectral density and gate–source bias.

Figure 7 depicts the channel current noise power spectral density as a function of the gate–source bias voltage at a drain–source voltage of 0.3 V and a temperature of 300 K. The figure illustrates that as the gate–source voltage increases, the trends of channel current noise and full shot noise remain largely consistent, which suggests that the noise in the channel is primarily dominated by shot noise. Between gate–source voltages of 0.60 and 0.75 V, the channel current noise closely aligns with the full shot noise, indicating that the suppression of shot noise is minimal at lower gate voltages. The channel current noise gradually increases when the gate–source voltage exceeds 0.75 V. This increase is attributed to the higher gate voltage, causing gate carriers to tunnel through the oxide layer, generating gate-tunneling shot noise and cross-correlated noise. Simultaneously, the increased channel current noise gradually deviates from the full shot noise. This deviation occurs because the increased gate voltage leads to a transition in the transport properties of the device from ballistic to quasi-ballistic [30,31,32]. The increased longitudinal electric field increases the carrier density in the channel, resulting in increased Coulomb and acoustic phonon scattering. The Coulomb interaction enhances the suppression of shot noise, thereby reducing channel shot noise and gate-tunneling shot noise in the channel region of the device.

**Figure 8:**
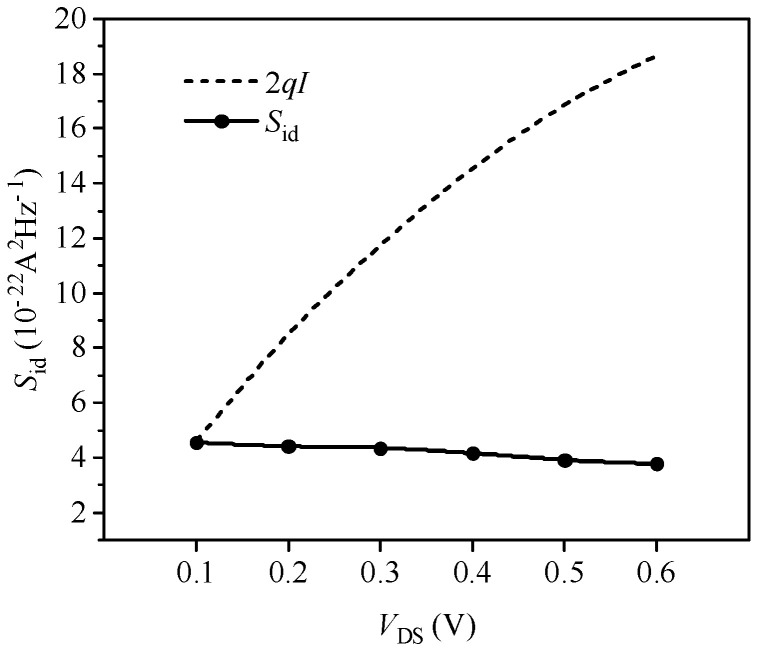
Relationship between simulation power spectral density and source–drain bias.

Figure 8 depicts the variation in channel current noise power spectral density with source–drain voltage under the conditions of the gate–source bias *V*_GS_ = 0.8 V and a temperature of 300 K. The figure illustrates that the channel current noise decreases gradually with increasing source–drain voltage, but the change is minor. Concurrently, the difference between the channel noise and the full shot noise gradually increases, indicating that the suppression intensity of shot noise is strengthening. This is due to the increase in source–drain voltage leading to a rapid rise in the transverse electric field, resulting in velocity saturation and strong electric field effects. These effects reduce carrier scattering in the channel, weakening the Coulomb interaction suppression of shot noise. Additionally, the increased drain barrier height enhances the Fermi effect suppression of shot noise. Due to the sufficiently small channel dimensions of the 10 nm MOSFET devices, the Fermi effect suppression of shot noise outweighs that of the Coulomb interaction; hence, the variation in channel current noise with the increase in source–drain voltage is not significant. These simulation results are consistent with experimental measurements of 10 nm MOSFETs reported in the literature [33,34].

### 3.6. Effect of Temperature and Doping Density on Channel Noise

**Figure 9:**
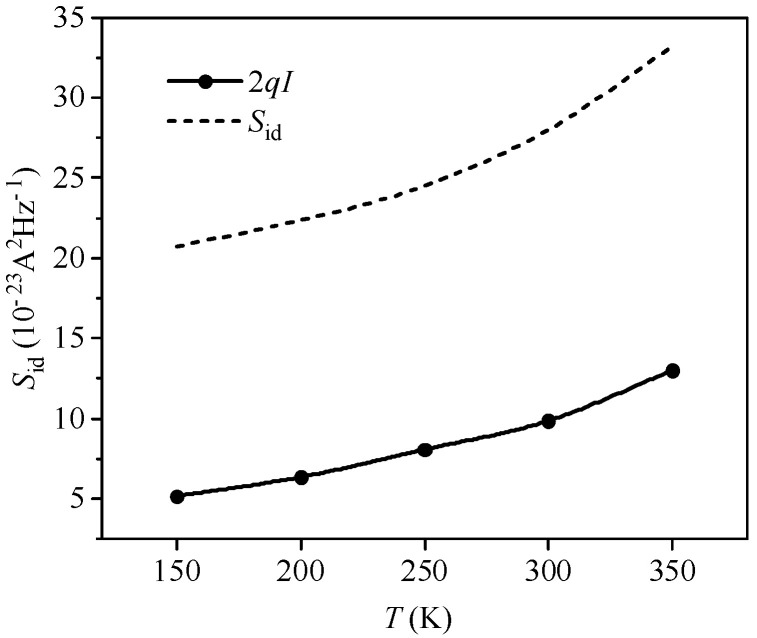
Relationship between simulation power spectral density and temperature.

Figure 9 illustrates the curve of the channel current noise power spectral density versus temperature at a gate–source bias *V*_GS_ = 0.75 V and a source–drain voltage *V*_DS_ = 0.3 V. The figure shows that as the temperature increases, channel current noise rises and shot noise suppression intensifies. In the low-temperature range (150–250 K), the number of carriers is low owing to incomplete impurity ionization. Meanwhile, the scattering mechanism of the device is mainly ionized impurity scattering. Its scattering probability is positively correlated with the density of ionized impurities and negatively correlated with temperature [35]. The average velocity of carrier thermal motion increases with temperature, enabling carriers to skim over the impurity ions faster with smaller deflection angles, making carriers less likely to be scattered. Thus, in the low-temperature range, the suppression of shot noise by the Coulomb effect is weak, and the channel current noise is primarily governed by the shot noise. From 250 to 300 K, the scattering mechanism in the device is primarily dominated by acoustic phonon scattering, which increases with temperature. The increased occurrence of acoustic phonon scattering enhances its suppressive effect on shot noise. Nevertheless, the channel current noise continues to increase, suggesting that the suppression of shot noise by the Coulomb effect is less effective in 10 nm channel devices compared to longer channel devices. Above 350 K, noise levels continue to rise due to increased ionized impurity concentration and carrier density, leading to higher current noise. This also indicates that in the channel noise of 10 nm MOSFET devices, thermal noise cannot be neglected.

**Figure 10:**
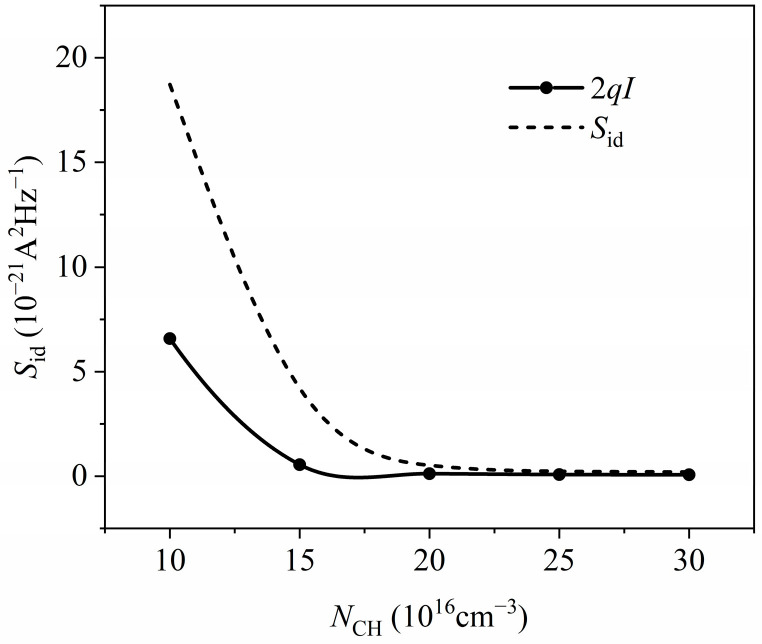
Relationship between simulation power spectral density and substrate doping density.

Figure 10 depicts the relationship between channel current noise power spectral density and channel doping density at a gate–source voltage *V*_GS_ = 0.75 V, a source–drain voltage *V*_DS_ = 0.3 V, and a temperature of 300 K. As the channel doping density increases, the channel current noise tends to decrease and approaches the full shot noise. This trend arises from two factors: First, a higher channel doping density results in more ionized impurities, which increases ionized impurity scattering and enhances the suppression of shot noise by the Coulomb effect. Second, as the channel doping density increases, the potential difference between the substrate and the source–drain contact increases, further suppressing shot noise via the Fermi effect. Thus, at room temperature (300 K), the primary component of channel noise in the 10 nm MOSFET remains to be the suppressed shot noise.

## 4. Conclusions

Simulation methodologies based on the drift–diffusion (DD) and hydrodynamic (HD) models are inadequate for capturing the noise performance of devices with channel lengths below 100 nm. This study introduces a 3D-MC simulator designed to simulate the drift and diffusion of real carriers in MOSFET devices. Using the MC simulation framework outlined in this study, raw data for the channel current noise of a 10 nm MOSFET device are simulated, and the channel current noise power spectral density is calculated. The simulations indicate that in 10 nm MOSFET devices, channel noise in the strongly inverted region is primarily characterized by suppressed shot noise and cross-correlated noise. The suppression effect is slightly enhanced with increasing gate–source bias voltage and source–drain bias voltage. Furthermore, analyzing the impact of temperature and channel doping density on channel current noise reveals that, at low temperatures, the 10 nm MOSFET device predominantly exhibits shot noise. As the temperature increases, the proportion of thermal noise in the channel current noise of the MOSFET device slightly increases. However, for the 10 nm MOSFET device, the main component of channel current noise remains the suppressed shot noise.

## Figures and Tables

**Table 1:** Main physical parameters.

Parameters	Numerical Value
$\mathrm{Phonon}\;\mathrm{Energy}\;\hbar \omega_{q}$	0.0579 eV
$\mathrm{Optical}\;\mathrm{Deformation}\;\mathrm{Potential}\;D_{0}$	10^8^ eV/cm
Material Defect Potential Σ	9.0 eV
$\mathrm{Intrinsic}\;\mathrm{Doping}\;\mathrm{Concentration}\;n_{0}$	1.5 × 10^10^ cm^−3^
$\mathrm{Electronic}\;\mathrm{Effective}\;\mathrm{Mass}\;m^{*}$	8.1254 × 10^−31^ kg
$\mathrm{Boltzmann}\;\mathrm{Constant}\;k_{B}$	1.380 × 10^−23^ J/K
Electrical Quantity q	1.602 × 10^−19^ C
$\mathrm{Elastic}\;\mathrm{Constant}\;c_{l}$	1.69 × 10^7^ N/cm^2^
Non−Parabolic Factor α	0.5 eV^−1^
Silicon Material Density	2.239 g/cm^3^
Silicon Forbidden Band Width	1.119 eV

## Data Availability

Data will be made available upon request.
